# Pre-Participation and Follow-Up Screening of Athletes for Endurance Sport

**DOI:** 10.14740/jocmr2129w

**Published:** 2015-04-08

**Authors:** Roman Leischik, Birgit Dworrak, Peter Foshag, Markus Strauss, Norman Spelsberg, Henning Littwitz, Marc Horlitz

**Affiliations:** a Faculty of Health, School of Medicine, University Witten/Herdecke, Germany

**Keywords:** Physical activity, Pre-participation screening, Athletes, Endurance sport

## Abstract

Physical activity increases life expectancy and sport is *a priori* not harmful. Exhausted sporting activity (e.g. endurance running, triathlon, cycling or competitive sport) can lead under individual conditions to negative cardiac remodelling (pathological enlargement/function of cardiac cavities/structures) or in worst case to cardiac arrhythmias and sudden cardiac death (SCD). This individually disposition can be genetically determined or behaviourally/environmentally acquired. Overall competitive young male athletes suffer five-fold higher than non-competitive athletes from sudden death and athletes aged over 30 bear a potential for arrhythmias, atrial fibrillation or a 20-fold higher possibility for SCD as female athletes. Patients with diabetes, coronary disease, obesity or hypertension require different special managements. Screening of cardiorespiratory health for sport activities has a lot of faces. Basically there is a need for indicated examinations or possible preventive measures inside or outside of pre-competition screening. The costs of screening compared to expenditure of whole effort for sporting activities are acceptable or even negligible, but of course dependent on national/regional settings. The various causes and possibilities of screening will be discussed in this article as basic suggestion for an open discussion beyond national borders and settings.

## Introduction

Basically, endurance sport can be recommended, because professional endurance athletes live longer than the general population [1-3]. Professional athletes have the advantage of pre-participation and follow-up screening before and during competitive sporting activity. If there is any suspicion on health problems, “unhealthy” athletes never will be a part of a professional team. Sharma et al [4] discussed the problems of pre-participation screening in young athletes mainly because of sudden cardiac death (SCD), but SCD is only one of many causes why we have to recommend a voluntary or mandatory screening in competitive athletes. In the case of a young athlete, SCD is with an incidence of 1:160,000/person/years [5] a rare and unexpected, but always tragic event. In the USA 50 - 75 deaths per year occur in young athletes, in France about 10 - 15 [6]. Marijon et al [6] describe the common risk of SCD in connection with sport with 5.4 up to 16.7/1,000,000/year, depending on the region. The mean age of the persons concerned was 46 ± 15 (11 - 75) years, 92% died directly during sport, only 12.7% had disorders before and 86.5% had a regular training. SCD occurred in young athletes with a frequency of 9.8/1,000,000/year, in young non-athletes with 2.2/1,000,000/year [6]. Among the general population the risk is about 9.2/1,000,000/1 year for men and 0.4/1,000,000/ year for women. Young competitive athletes (Table 1) have a five-fold higher risk than non-competitive athletes and men have a 20-fold higher risk than women. More attention should be paid to the variety of causes than to the absolute figures, which may vary widely over the years among the authors [6-10] (Table 1). Cardiac death related to sport occurred in most cases during sporting activity or within 1 h later and is mainly related to a disbalance of oxygen demand and supply [11]. Literature differentiates between “young” and “old” athletes (< 35 and > 35 years). This classification is of historical origin, not logically explained and based on publication of Thieme et al [12]. It is not reliable, because more than two-thirds of SCD due to myocardial infarction (beyond of sport) occurred in the age group of 30 - 35 years [10] (in persons aged 5 - 35 years). In athletes until 35 years, Solberg et al [8] found in 48% of all cases of sudden death a coronary disease as the main possible cause of SCD. Classification in age groups < 35 and over > 35 years includes also coronary disease as cause of SCD to both groups. So far, depending on age and country, there are significant differences in pre-competition screening [5, 9]. Sharma et al [4] discussed pitfalls of electrocardiographic (ECG) screening, which was valuable in Italy [13], but with low predictive value in Britain [14]. AHA screening recommendations without basic ECG might have similar impact for outcome regarding SCD as Italian experience with ECG [15]. Use of echocardiography as screening [16] method before participation in sport was controversial discussed by Sharma et al [4] without clear recommendation. This problem is not new. Generally, it seems to be a problem to go to an official step further and classify “American” or “Italian” way of athletes screening as history.

**Table 1:** Distribution of Cardiovascular Causes of Sudden Death in Young Athletes > 12 - 35 Years and General Population of Young People 5 - 35 Years (in %)

	Marijon et al, 2011 [6]	Corrado et al, 2003 [7]	Solberg et al, 2010 [8]	Maron et al, 2007^9^	Puranik et al, 2005 [10]
Aortic rupture/dissection	2	1.8	4.3	2	5.4
Aortic stenosis/cong HD	6		4.3	5	
Arrhythmia					29
ARVC	4	22		4	1.6
Channelopathies (QT, WPW)	12	1.8	8.7	3	(29?)
Coronary artery anomalies		11	3.3	17	2.1
Coronary disease	6	18	48	3	24.5
Dilatative CM	4	1.8		2	5.4
Hypertrophic CM	10	1.8	4.3	36	5.8
MVP	2	7.3		4	
Myocarditis	4	9	22	6	11.6
Possible HCM	4			8	
Riva muscle bridge	2	3.6		3	
Unclear	36	1.8			
Other (endocarditis, clots, etc.)					7.5
	n = 50	n = 55	n = 22	n = 1,435	n = 241

cong HD: congenital heart disease; ARVC: arrhythmogenic right ventricular cardiomyopathy; QT: QT-syndrome (including Romano-Ward syndrome and Jervell-Lange-Nielsen syndrome); WPW: Wolff-Parkinson-White syndrome; CM: cardiomyopathy; HCM: hypertrophic cardiomyopathy; MVP: mitral valve prolapse.

All recommendations for screening (as sport itself) are voluntary.

The previous official concepts include a physical examination and medical history of the young athlete and in the Italian version additionally a resting-ECG. These concepts may have been sufficient in the past for young athletes, but are no longer sufficient considering today’s knowledge and extensive competitions. Compared with the investment of time and money [17] in sporting activities, the previously recommended measures represent the strict minimum. Based on the experience of last 20 years in daily sports medicine practice, different causes of athletes screening could be defined.

## Methods of Screening

### Resting-ECG

**Figure 1:**
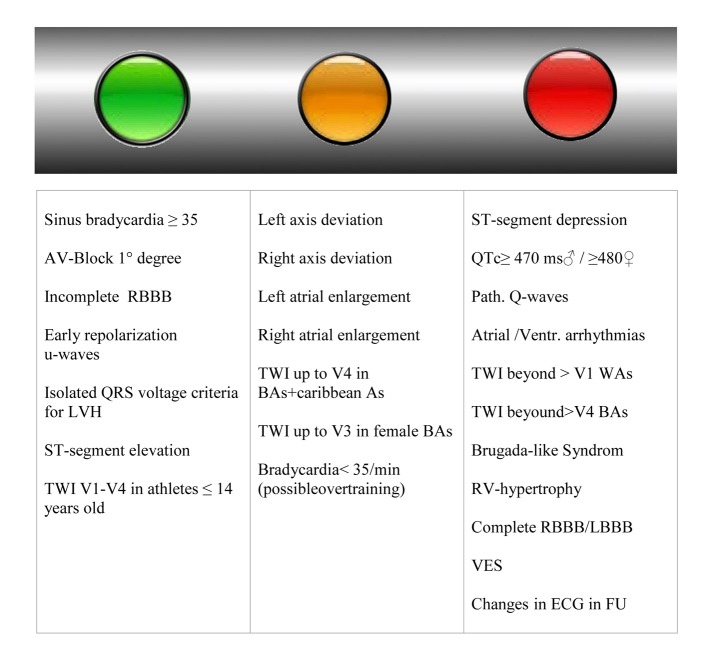
Green light: no strict recommendation [14, 21-23] for further examination (in our opinion minimum echocardiography is recommended). Yellow light: borderline changes, potentially further recommendation for evaluation. Red light: further evaluation strong recommended. RBBB: right bundle brunch block; LVH: left ventricular hypertrophy; TWI: T-wave inversion; BAs: black athletes; WAs: white athletes; LBBB: left bundle brunch block; RV: right ventricular; FU: follow-up; VES: ventricular extrasystole.

We have a variety of findings and possibilities for cardiac sudden death [3, 9, 10], which cannot be diagnosed by simple 12-channel-ECG. ECG is an orientating examination, without correlation to hypertrophy [14, 18] and information about the dimensions of heart cavities. Forty percent of the athletes show abnormal changes in their ECGs [19]. The frequency of ECG-changes depends on ethnic origin, as well as on type and intensity of training and kind of sport. Here, mainly changes such as prolonged QRS-complexes, diverse changes in T-waves (negativities), deep Q-waves or even “bizarre” ECGs are recorded [20]. Changes in ECGs should be analyzed according to “Seattle criteria” [21] or to the European recommendations [14, 22, 23]. Ethnic differences in repolarization changes should be considered [14, 24]. QT-segments > 470 ms [14, 22] always require an individual approach and at least anamnestic-familiar or even genetic examinations. There is always a need of careful monitoring of ECG-changes and course. ECG-changes are described as a “traffic light scheme” in Figure 1 in terms of their significance. Sinus bradycardia < 35/min can be a sign of overtraining.

### Exercise test

**Figure 2:**
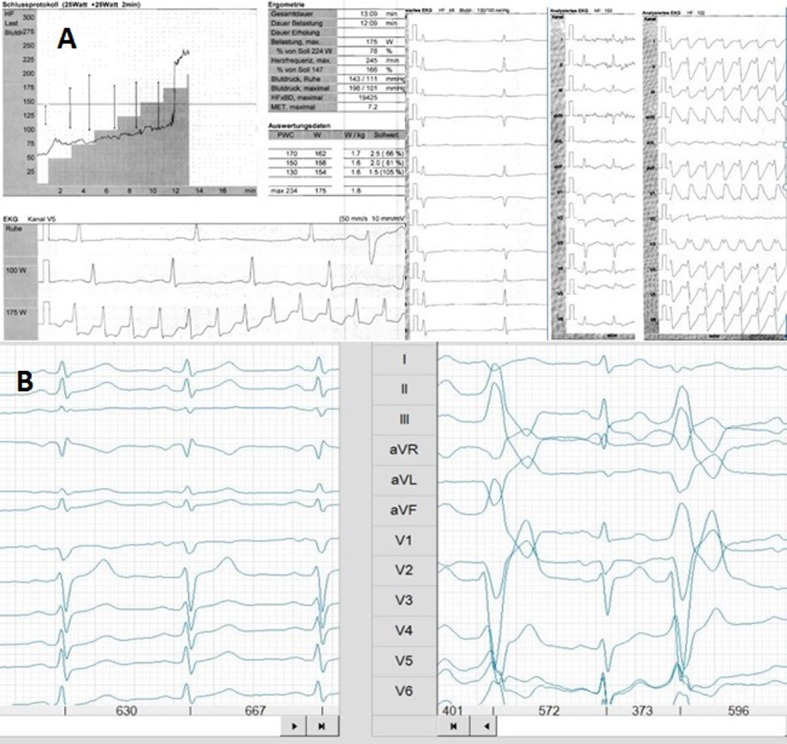
Exercise ECG shows onset of atrial fibrillation in “healthy” athlete (A) or ventricular ectopic beats (B) during exercise (normally “healthy” athlete).

Exercise ECG (EECG) is a relative old method with low sensitivity for one vessel coronary heart disease (CHD) [25] and with false positive findings in athletes [26, 27]. The advantage is, EECG is easy to perform (use of educated technicians) and offers basic performance data (heart rate, blood pressure values, oxygen (O_2_) saturation), a clear information about arrhythmias during exercise (Fig. 2) or exercise induced hypertension [28] and load performance in watts. EECG can be combined with lactate measurements or spiroergometry. In case of abnormalities during EECG it can be combined with physical stress echocardiography [29] in suspicious coronary disease (normally all athletes can be examined using physical stress). We prefer a special stress echocardiography chair [29].

### Echocardiography

**Figure 3:**
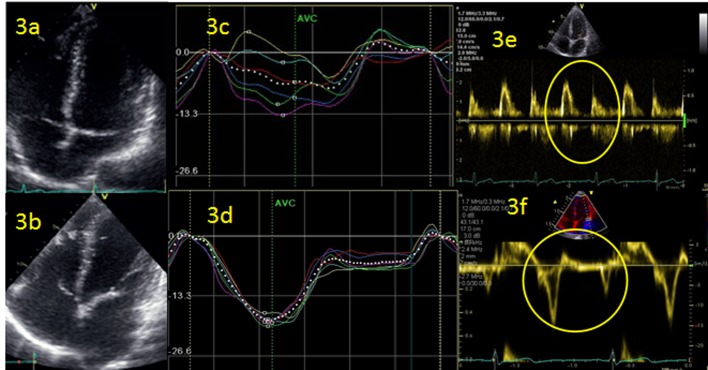
(a, b) Left atrium (LA) follow-up 2 years of triathlete with enlargement of LA and atrial fibrillation events. (b, c) Strain curves of left ventricle during atrial fibrillation (c) and 24 h after recovery in sinus rhythm (d). (e, f) Doppler measurement of diastolic function: conventional (e) and using tissue Doppler imaging (TDI).

Changes in the heart-valves of an athlete (acceleration of the extent of stenosis in the elderly), aortic dilatation [30], and enlargement of the atriums [28] (Fig. 3a, b) represent only a few of the changes that are easily detectable by echocardiography. Functional changes can be excellently investigated by Doppler, tissue Doppler imaging (TDI) or strain-technique [31, 32]. In fact, the FIFA has taken a lead [33] in the respect of recommendations and postulates a well-founded echocardiography, based on the publications of Dvorak et al [34] and Thunenkotter et al [35, 36]. FIFPro fights for national and international standards in pre-competition screening of football players (young and old) [37]. Echocardiography carried out in an athlete provides together with the description of cardiac structures (extent of hypertrophy [28, 38], aorta [30], atrium [39], dimensions of ventricle [28, 40], structure of myocardium, and wall thickness [28]) also functional information [32]. Doppler measurements give information about the blood flow rate, diastolic function [41], segmental velocity (TDI) (Fig. 3d, e) and strain-technique offers segmental and global myocardial or atrial [42] values/curves (Fig. 3c, d) of deformation within the cardiac cycle [31, 32, 43]. The follow-up examinations are very important in cases of drop of power or myocarditis. In cases of competitive young athletes, which should train and compete at maximal exercise level the decision to decline the special discipline in case of mitral valve prolapse, aortic root dilatation, pathological hypertrophy can be easier (reserved for an experienced sport cardiologist).

### General situation for screening in sport

Not only SCD represents a kind of complication due to sport, but also structural changes of the heart (aorta, atrium, right or left ventricle) and possible arrhythmias.

In daily routine ambitious amateur and competitive professional athletes represent only 50% of the patients of a cardiologic practice focused on sports medicine (internal data). Drop in performance, hypertension [28], atrial fibrillation [44] and possible coronary disease [45] rather dominate the clinical spectrum in the case of ambitious amateur athletes. Occurrence of atrial fibrillation can alter an active and quasi-healthy leisure-athlete to a physically non-active athlete. In the other 50% of amateur athletes medical work consists in performance assessment and determining indications for measures of exercise if CHD, obesity, arterial hypertension or metabolic syndrome is present or to maintain physical abilities in the elderly. Here optimization of performance or competition preparation plays a minor role.

However, CHD is relatively common in young athletes < 35 years [8]; accordingly also younger athletes should undergo exercise tests. In cases of abnormal ECG-findings a stress echocardiography should be carried out [29]. Athletes > 30 years of age, undergoing an extreme endurance competition, for example triathlon or marathon, should be examined by stress echocardiography from a prognostic point of view. Competitive ambitious athletes < 35 years should undergo a specific cardiologic examination (including basically echocardiography and exercise test) and regular annual check-up examinations if problems occur and before starting a training. When myocarditis, fibrosis or right-ventricular dysplasia is suspected, cardio-MRT examination is recommended [46, 47].

### Current situation of screening and causes of preventive medical care in daily routine of sports medicine

Previous official recommendations are subject of constant change [30, 34-36, 48, 49] and should follow the recommendations for patients with CHD [50] and new knowledge/experience [34-36]. The following recommendations for screening are based on active care of competitive and amateur athletes since 20 years and own sports experience. In principle, the following four reasons for examination can be distinguished: 1) screening-examination (baseline examination); 2) general preventive check-up (periodical follow-up examinations); 3) check-up in the case of discomfort (loss in performance, dyspnoea, thoracic pain, and infection); 4) performance diagnostics for training recommendation.

#### Screening-examination (baseline examination)

The baseline examination includes, in addition to the physical basic examination and medical history, the following tests: echocardiography, EECG and pulmonary function testing as well as a basic blood test (differential blood count, CRP, creatinine, GPT, GOT, gamma-GT, LDH, LDL-/HDL-cholesterol, triglycerides, blood glucose, iron, ferritin, TSG, and electrophoresis); furthermore (athletes > 35 years) a duplex sonography of carotids [51, 52] (determination of intima-media thickness, plaques) is carried out in order to calculate the state of atherosclerosis. In case of abnormal findings in resting or EECG a stress echocardiography is performed. Within echocardiography all modern examination techniques are applied, including strain-echocardiography. Using echocardiography nowadays not only the extent of hypertrophy, the size of aorta, ventricles and atriums and the functioning of cardiac valves as well as the visual contractions can be analyzed and recorded, but also complex measurements of function in the area of both ventricles and atriums can be carried out by means of TDI, strain technology [32, 53] as well as conventional CW-/PW-Doppler for systolic and diastolic blood velocities [54]. Thus, changes, particularly over the course, can be registered. These, for a non-cardiologist, highly complex echocardiographic examinations have now become routine for an experienced cardiologist. As standard, we offer a spiroergometry to athletes, in order to document the physiological performance profile [40] (amongst others threshold of fat burning, aerobic capacity, anerobic threshold) and to estimate the maximum oxygen uptake (VO_2_ max in mL/min/kg) [40, 55]. Optional the body composition might be documented by means of more complex impedance scales [56].

#### General preventive check-up

The usually annual carried out check-up includes always an echocardiography [40] and a performance test [35, 40, 55]. We attach particular importance to the documentation of hypertension [28, 35] during exercise and documentation of heart rate changes or premature heart beats. Echocardiography is conducted mainly to compare the size of ventricles/atriums and to detect possible changes in heart valves and aorta or to assess myocardial hypertrophy. A dilatation of the aorta with consecutive aortic insufficiency is not a rare event in the case of an endurance athlete (2%) [40]. Ambitious and competitive athletes are often examined “off-season” by spiroergometry, to check their training schedule. That is also the case for competitive athletes in their pre-competition season.

#### Check-up in the case of discomfort or drop of performance

Here, the examination is focused on the clinical situation. Overtraining should be considered. A drop of performance can have a lot of causes. In most of these cases, the health situation of a young, old, leisure, ambitious or competitive athlete is very complex and may claim a wide interdisciplinary range from immunology, cardiology, orthopedics up to orthodontics.

All new changes in resting-ECG, syncope, or a drop of performance need to be clarified consequently. In addition to the basic examination, including echocardiography and resting-ECG, all further examinations are carried out to the clinical demand (stress echocardiography, Holter-ECG, Angio-CT [57], cardio-MRI [46, 47] (myocarditis/fibrosis) up to coronary angiography and electrophysiological examination). A detailed blood analysis as described above and additionally antibodies and hormones (e.g. on account of inflammation or hormonal changes), including the determination of virus antibodies (Epstein-Barr, Cytomegaly, Herpes, Parvo, Echo, Coxsackie, etc.) and bacterial antibodies (*Chlamydia pneumoniae* [58] and Borrelia), should be considered in case of a drop of performance. Negative findings of virus-antibodies do not exclude a myocarditis. Further step in the diagnostic is cardiac-MRI and a myocardial biopsy (in cases of reduced cardiac function). With this approach, so far we have not had any case of death or undetected myocarditis since 18 years. In cases of possible coronary disease, stress testing/EBT/CT-angiography [57] or coronary angiography should be considered.

#### Performance diagnostics

Diagnostics by means of spiroergometry [40, 59] is extremely helpful planning the training [40, 55, 60] and specifying the performance level of active [40]/professional [55] athletes and planning activities of affected patients [61, 62]. In hobby-athletes/patients with metabolic syndrome or diabetes [61] a spiroergometry is carried out, to check the current performance level and to plan an aerobic training schedule. The focus lies here on fat burning and careful planning of activities in the aerobic zone [62].

## Conclusions and Perspectives

The cost-benefit ratio of a combined exercise-ECG and echocardiography or spiroergometry and echocardiography in terms of the costs of an ambitious sport activity or competitive sports is acceptable. Particularly by participation of the public German health insurance system in the costs, the medical care of the athletes here is ensured [63]. But also in other countries, the costs for screening-examinations should be regarded as negligible, given the high expenditures for preparation and participation in marathon and triathlon competitions [17] or intensive costs in professional football [64] and other team sports [65].

Regarding all the competitive sporting activities with an enormous importance for hobby- and professional athletes (e.g. FIFA [33]), media and industry, physical activity in general population is of fundamental importance [16, 66]. Accordingly, prevention of sudden sport-related deaths or a “negative remodeling” by sport has not only an individual component, but also a significant social impact on physical activity in general population. In this regard, further expenses, studies and well-founded pre-competition screening in the industrialized world are socially justified and financially reasonable. Long-lasting dispute with many publications about use of resting-ECG or not, in pre-competitive sport screening belongs to the past. A small price-echocardiography in German social insurance medicine (about 40$) can serve as template and exercise test should be not only reserved for professional teams. Inequalities in sport screening should not be a cause for natural selection or later complications of aortic/atrial enlargement and arrhythmias. These complications can be seen in centers with an opportunity for long-term care with long-term follow-up.
